# Aging differentially affects online control and offline control in finger force production

**DOI:** 10.1371/journal.pone.0198084

**Published:** 2018-05-31

**Authors:** Yang Sun Park, Kyung Koh, Hyun Joon Kwon, Okjin Lee, Jae Kun Shim

**Affiliations:** 1 The Department of Physical Education, Hanyang University, Seoul, Republic of Korea; 2 The Movement Science Center of Research Institute for Sports Science and Sports Industry, Hanyang University, Seoul, Republic of Korea; 3 The Department of Kinesiology, University of Maryland, College Park, MD, United States of America; 4 The Department of Mechanical Engineering, College of Engineering, Kyung Hee University, Yong-in, Republic of Korea; 5 The Department of Sports & Leisure Studies, Kwangwoon University, Seoul, Republic of Korea; University of Georgia, UNITED STATES

## Abstract

Human central nervous system (CNS) undergoes neurological changes during the aging process, leading to declines in hand and finger functions. Previous studies have shown that the CNS can independently process multi-finger force control and moment of force control. However, if both force and moment control are simultaneously imposed by motor task constraints, the CNS needs to resolve competing interests of generating negative and positive covariances between fingers, respectively, which causes “*conflict of interest or COI”*. Here, we investigated how aging affects the CNS’s abilities to solve *COI* through a new experimental paradigm. Both elderly and young subjects performed a constant force production task using index and middle fingers under two conditions, multi-finger pressing with no *COI* and with *COI*. We found that the elderly increased variance of a virtual finger (VF: an imagined finger producing the same mechanical effect as both fingers together) in time-to-time basis (i.e. online control), while increasing covariance between individual fingers (IF) forces in trial-to-trial basis (i.e. offline control) with *COI* than *no COI*. Aging affects the CNS’s abilities to solve *COI* by deteriorating VF actions in online control and IF actions in offline control.

## Introduction

Our hands are one of the most versatile parts of the human body, and we use them to perform a variety of day-to-day activities such as eating, writing, driving, etc. During aging, the hands undergo many neurological and biomechanical changes, negatively impacting hand dexterity and consequentially the quality of life in the elderly [1, 2]. Previous studies have shown that aging is associated with the declines in strength [3], muscle mass [4], and finger dexterity [5] as well as degeneration of the central nervous system [6]. However, the effect of aging on the CNS’s control mechanisms of hand function is still poorly understood, specifically regarding the association between aging and external task constraints that are imposed by motor tasks [7].

Previous studies have shown that multi-finger actions are controlled in a hierarchical manner with at least two levels in the hierarchy: the actions of individual fingers (IF) at the lower level and the actions of the virtual finger (VF: an imagined finger producing the same mechanical effect as all fingers together) at the higher level [8–11]. Recently, the hierarchical variability decomposition (HVD) model was introduced for quantification of the hierarchical organization of multi-finger actions [12]. The HVD model decomposes variability in the motor system into mathematically independent components, each of which quantifies distinct motor behaviors. In the HVD model, *estimatibility*, *consistency*, and *repeatability* are quantified at the VF level [13]. In a constant force production task using multiple fingers, *estimatibility* reflects the CNS’s ability to estimate the target force, and *consistency* reflects the CNS’s ability to consistently perform the task on a moment-to-moment basis (i.e. online control), while *repeatability* reflects the ability to reproduce the same behavior on a trial-to-trial basis (i.e. offline control) over multiple trials at the higher level. The consistency and repeatability can be further decomposed into *workspace* of multiple fingers and multi-finger *synergy* between the multiple fingers at the lower level (or IF level). For the constant force production task by multiple fingers mentioned above, *workspace* is quantified as the sum of variances created individual motor effectors or fingers, which indicates a multi-finger force space utilized by the CNS to perform a particular task with multiple fingers. *Synergy* is quantified as covariances between multiple finger forces [12, 13], which reflect the CNS’s control strategies to utilize motor effectors within the *workspace* for a particular purpose (i.e. *consistency* and *repeatability*). Here, the current study intends to investigate how aging affects these dependent variables during a multi-finger action using the HVD model.

The concept of multi-finger synergy has been introduced as a critical aspect of hand control mechanisms during multi-finger actions [14]. During static grasping of a hand-held object, the CNS produces grasping forces and achieve the ‘‘linear equilibrium” as well as moments of forces that to achieve the ‘‘rotational equilibrium” of the hand-held object. Stability of linear and rotational equilibrium required in our experimental paradigm involving a miniature “seasaw” is similar to grasping a free object statically. It has been suggested that the CNS generates two types of multi-finger synergies, force-stabilizing synergy for stability of grasping and moment-stabilizing synergy for rotational equilibrium [10–12]. For example, if one is asked to produce a constant pressing force with index and middle fingers, the CNS may negatively co-vary two finger forces so that the VF force, the sum of two-finger forces, can be stabilized resulting in more consistent VF outputs. This has been referred to as force-stabilizing synergy. Similarly, if one is asked to produce a constant total moment of force (VF moment) on a miniature “seesaw” for the stabilization of rotational equilibrium, two finger forces need to be positively co-varied for the purpose of the VF moment stabilization assuming that the moment arms are constant. This has been referred to as moment-stabilizing synergy. Previous studies have suggested that these two types of synergies are controlled independently by the CNS, which is consistent with the principle of superposition originally suggested in robotics [11, 15, 16].

Age-related changes in dual-task paradigm have been favorite topic in the last decade. Much attention has been paid to several aspects of dual task performance such as reaction time [17], performance parameters of gait [18] or postural control [19]. However, it is little known about how aging affects the CNS’s control mechanisms in multi-finger actions. In particular, when two tasks impose constraints that are conflicting each other in a particular dual task, the CNS needs to resolve the competing interests. This dual task creates “*conflict of interest or COI”* to the CNS, which needs to perform the task with a solution. A recent study on constant force production using four fingers has demonstrated that the young people can manage the *COI* problem while showing unchanged force-stabilizing synergy when moment-stabilizing task was added as a secondary task [20]. This result suggests that the CNS of young people can successfully form force-stabilizing synergy between fingers when performing a motor task with two sub-tasks that are competing each other. However, it is currently unknown how this CNS ability to manage two conflicting sub-tasks is affected over the process of aging, even though previous studies found that aging was associated with deterioration of force-stabilizing synergy [21] and decline in motor performance in double-task [22].

The aim of this study was to investigate aging-related changes in the hierarchical organization of multi-finger actions under two conflicting task-constraint conditions. In order to achieve this aim, we asked subjects to perform a constant force production task while pressing force sensors mechanically fixed on a stationary frame with index and middle fingers. In another condition, an additional constraint was introduced by requiring a subject to produce a constant moment of finger forces on a miniature seesaw that had a fulcrum between two fingers. Considering other previous studies which demonstrated decreased performance in motor tasks with additional task constraints in older adults [17, 23], it was hypothesized that aging would be associated with deterioration in motor performance when an additional task constraint is introduced. Consistent with the previous study mentioned above [20], it is also hypothesized that younger adults would be capable of generating the force-stabilizing synergies even when an additional constraint is presented.

## Methods

### Participants

Fifteen young (8 male and 7 female) and fourteen elderly (5 male and 9 female) subjects participated in the study. The young group’s age, height, and body mass were respectively: 21.13 ± 1.35 years, 171.57 ± 8.43 cm, 70.29 ± 16.77 kg, while the elderly group’s age, height and mass were 79.13 ± 5.06 years, 157.73 ± 8.83 cm, 64.57 ± 7.03 kg. All subjects provided their written informed consent. The study was approved by University of Maryland, College Park Institutional Review Board.

### Experimental procedures

**Fig 1:**
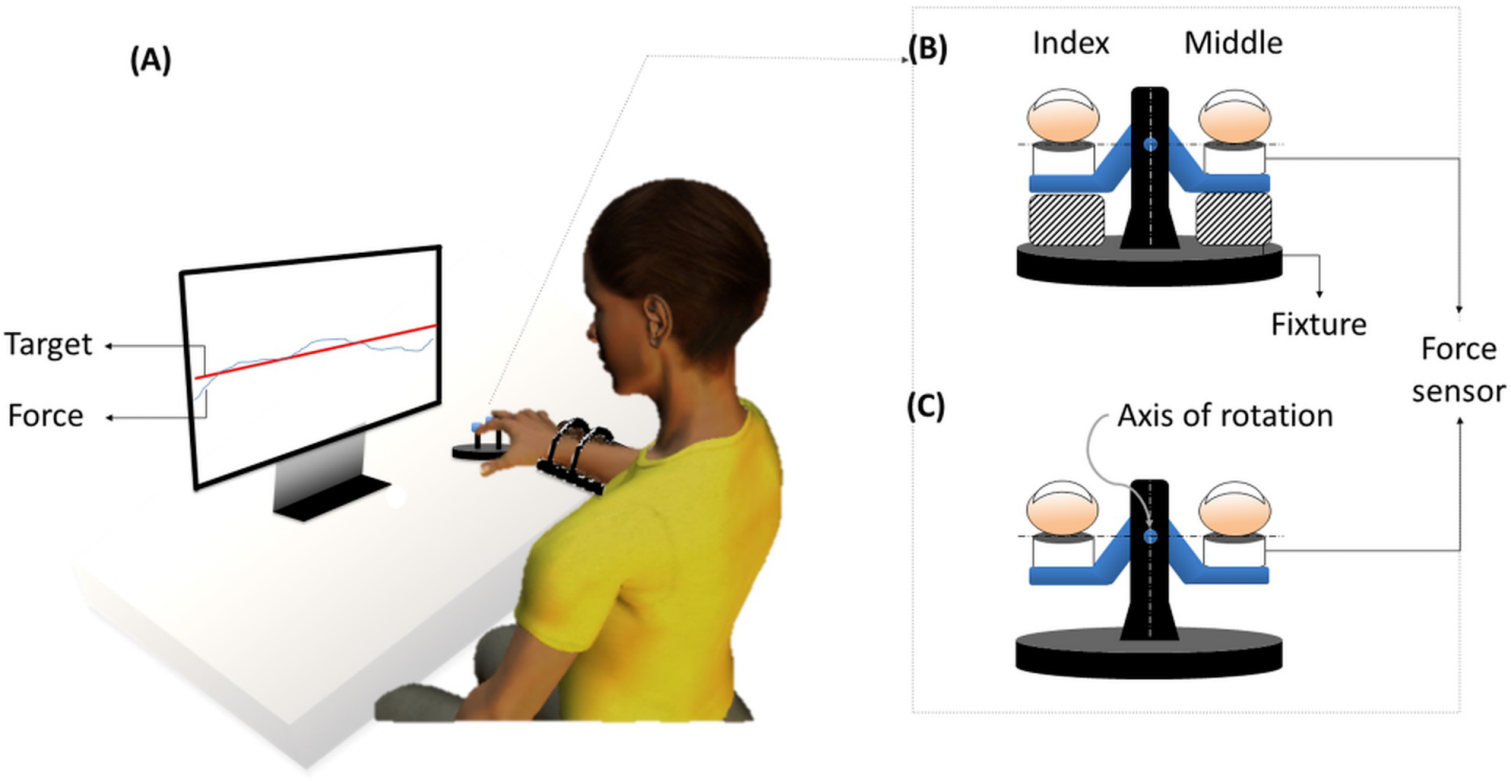
Schematic illustration of the experimental setup and experiment. (A) Subjects were asked to use index and middle fingers of the right hand, and press the force sensors at finger tips while matching the two-finger force sum (virtual finger or VF force) to 10N for 10 seconds over 20 trials for each of two conditions, with no additional constraint (NC) and with an additional constraint (AC) conditions. (B) NC condition: two plastic blocks were placed under the seesaw structure, which prevented a rotation of the experimental setup during the pressing task and (C) AC condition: the plastic blocks were removed, which allowed free rotation of the seesaw structure.

Subjects were asked to rest the distal phalanges of each of the two fingers of the right hand on force sensors (Models 208 M182 and 484B, Piezotronics, Inc., Depew, NY), such that all joints were slightly flexed and the hand formed a dome shape (Fig 1A).

Each subject was asked to produce a constant force of 10 N using the right index and middle finger for 10 seconds, which consisted of 20 trials per task. Subjects were shown the sum of the produced finger forces along with the 10 N target force line (Fig 1A). The task was performed under two conditions; with no additional constraint (NC) and with an additional constraint (AC). For the NC task, subjects were instructed to produce 10N force using index and middle fingers on fixed flat manipulandum (Fig 1B) and match the sum of the index (F1) and middle (F2) forces to the constant 10 N target force, satisfying the task equation, F1+F2 = 10N. For the AC condition, subjects were instructed to produce and match the constant 10 N target force using index and middle finger on a flat manipulandum which can freely rotate between two fingers about the anterior-posterior axis (Fig 1C) while keeping the rotational equilibrium, satisfying two task equations, F1+F2 = 10N (i.e. NC task) and F1 = F2 (i.e. AC task). The “seesaw” manipulandum was designed in such a way that the height of the axis of rotation was located at the same height as the contact surface of index and middle fingers with the force sensors in order to minimize the moments caused by shear forces of the fingers. When F1 and F2 are constant, that first and second constraints do not create a conflict. However, when the forces are dynamically changing as shown in any force control studies on humans, these two constraints create a conflict. For example, when F1 increases, F2 needs to decrease for force stabilization and F2 needs to increase for torque stabilization. The CNS needs to resolve these competing interests of generating negative and positive covariances (i.e. synergies) between fingers, respectively, which causes “*conflict of interest or COI”* in terms of formation of multi-finger synergies.

For each trial, force data from index and middle fingers were collected for 10 seconds and the last 5 seconds were used for analysis where the force production was at a steady state. The virtual finger (VF) force was calculated as the sum of two time-varying forces of index and middle fingers (or individual finger: IF). Using HVD model, the VF force for trial *i*, yi(t), was modeled as the sum of three components which reflect distinct motor abilities [12]:

$$y_{i}\left(t\right)=X_{i}\left(t\right)+E_{i}+m$$ (Eq. 1)

where *X*_*i*_*(t)* is demeaned VF force for trial *i*, *m* is the mean VF force after averaging over all timesteps of all 20 trials, and *E*_*i*_ is the difference between the mean VF force for trial *i* and *m*.

Online, *X*_*i*_*(t)*, offline components, *E*_*i*_, overall mean, *m*, extracted from VF force are mathematically independent. Online, *X*_*i*_*(t)*, and offline, *E*_*i*_, signals were further analyzed such as such as consistency, repeatability, and synergy to reflect the CNS’s control ability in online and offline controls, respectively. Overall mean, *m*, was analyzed further to refer to the CNS’s *estimability* [12, 13].

The motor task error was quantified as the overall mean-squared error (*OMSE*), the averaged squared deviation of the VF force from the 10-N target force:

$$OMSE=\frac{1}{N}\sum_{i=1}^{N}\left\{\frac{1}{\tau }\int {\left[f_{T}-y_{i}\left(t\right)\right]}^{2}dt\right\}$$ (Eq. 2)

Where τ is the 5-s duration of $y_{i}(t)$ for each trial, *f*_T_ is the 10-N target force, and N is the number of trials (N = 20).

**Fig 2:**
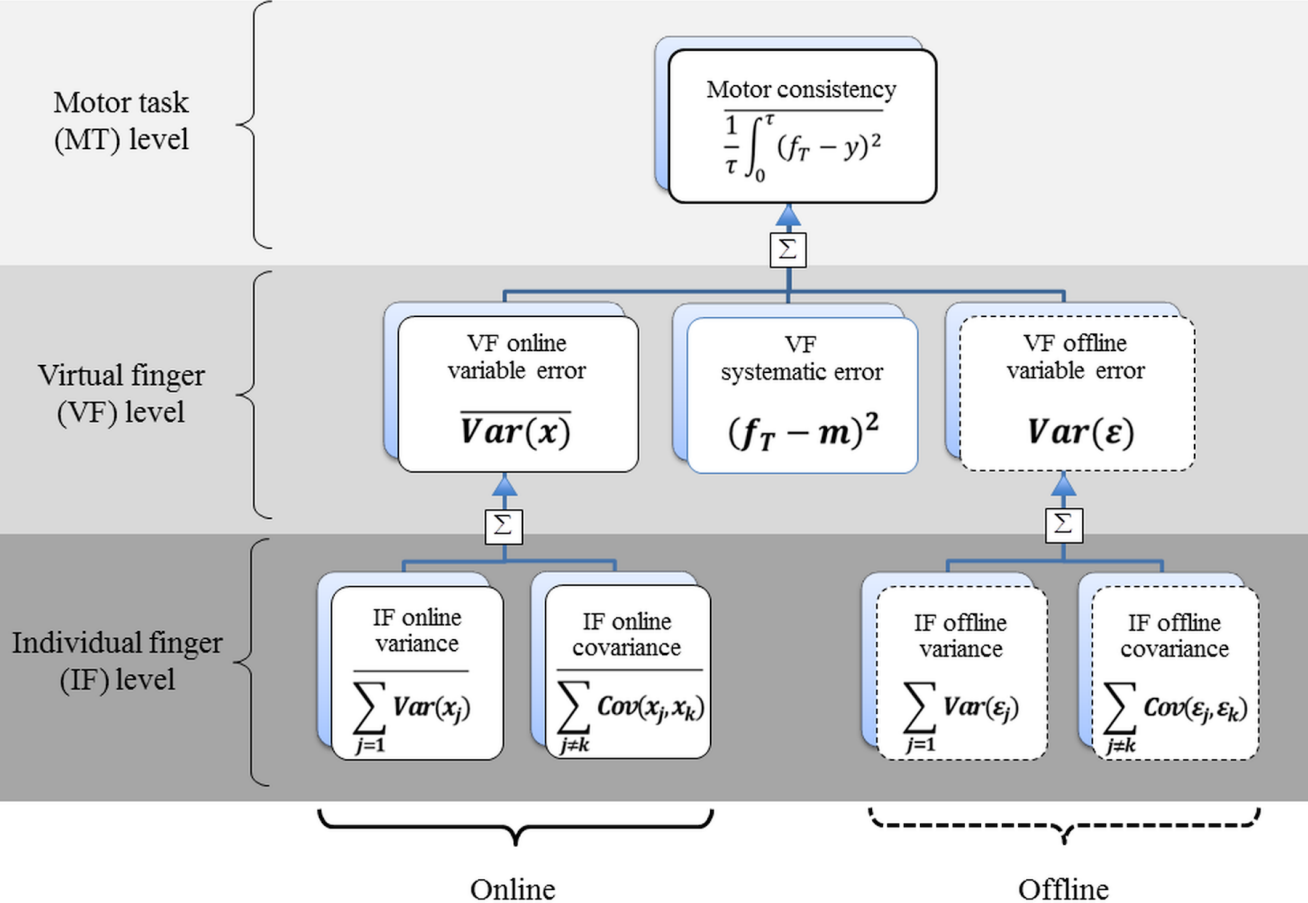
Hierarchical organization of multi-finger force variability in a redundant hand system. The overall mean squared error ($\overset{-}{\frac{1}{\tau }\int {\left(f_{T}-y_{i}\left(t\right)\right)}^{2}}$) is composed of or the linear sum of the intra-trial moment-to-moment (online) variable error ($\overset{-}{Var\left(X\right)}$), the trial-to-trial time-averaged (offline) variable error ($Var\left(E\right)$) and the systematic error (${\left(f_{T}-m\right)}^{2}$) at the virtual finger (VF) level where the task is performed with the sum of two finger forces (VF force). The online and offline variable errors at the VF level are composed of or the linear sum of individual finger (IF) force variances ($\overset{-}{\sum_{j\;=\;1}^{n}Var\left(x_{j}\right)}$ and $\sum_{j\;=\;1}^{n}Var\left(\varepsilon_{j}\right)$) and between-finger force covariances ($\overset{-}{\sum_{j\neq k}Cov\left(x_{j},x_{k}\right)}$ and $\sum_{j\neq k}Cov\left(\varepsilon_{j},\varepsilon_{k}\right)$) at the IF level.

The HVD model decomposes *OMSE* into several mathematically independent components in a hierarchical manner (Fig 2). In the VF level, *OMSE* was decomposed into three error components:

$$OMSE={VE}_{ON}+{VE}_{OFF}+SE=\;\overset{-}{Var\left(X\right)}+Var\left(E\right)+{\left(f_{T}-m\right)}^{2}$$ (Eq. 3)

where *VE*_*ON*_ is the “online variable error”, defined as the variance within a trial, averaged over all trials ($(\bar{Var\left(X\right)}$), *VE*_*OFF*_ is the “offline variable error”, defined as the variance between trials (*Var(E))*, and *SE* is the “systematic error”, defined as the squared overall deviation ((*f*_*T*_−*m*)^2^).

The online and offline variable errors quantify the moment-to-moment *consistency* and trial-to-trial *repeatability* of the motor task, and the systematic error is overall deviation of VF force from the target force (i.e. *estimability*) [13].

The online and offline variable errors are calculated as follows:

$$\overset{-}{Var\left(X\right)}=\;\overset{-}{Var\left(\sum_{J=1}^{n}x_{j}\right)}$$ (Eq. 4)

$$Var\left(E\right)=Var\left(\sum_{j=1}^{n}\varepsilon_{j}\right)$$ (Eq. 5)

where *x*_*j*_ is demeaned force of *j*^th^ finger, *ε*_*j*_ is the differences of *j*^th^ finger between the mean across time for each trial and the mean across all time steps and all trials, and *n* = 2 is the number of task fingers. The overhead bars indicate means over trials.

In the IF level, the online and offline variances were further decomposed as the sum of IF variances (*Var*_*ON*_, *Var*_*OFF*_) plus between-finger covariances (*Cov*_*ON*_, *Cov*_*OFF*_) (Fig 2):

$${VE}_{ON}={Var}_{ON}+{Cov}_{ON}=\overset{-}{\sum_{j=1}^{n}Var\left(x_{j}\right)}+\overset{-}{\sum_{j\neq k}Cov\left(x_{j},x_{k}\right)}$$ (Eq. 6)

$${VE}_{OFF}={Var}_{OFF}+{Cov}_{OFF}=\;\sum_{j=1}^{n}Var\left(\varepsilon_{j}\right)+\sum_{j\neq k}Cov\left(\varepsilon_{j},\varepsilon_{k}\right)$$ (Eq. 7)

The sum of IF variances, *Var*_*ON*_ and *Var*_*OFF*_ reflects the total amount of variability in the motor task (i.e. *workspace*), while the sum of IF covariances, *Cov*_*ON*_ and *Cov*_*OFF*_ reflects synergistic actions between finger forces (i.e. *synergy*) to attenuate or amplify the VF force through negative covariance or positive covariance between fingers, respectively [13].

### Statistical analysis

Standard descriptive statistics were used: the data are presented as means ± standard errors (SE). Two-way mixed analyses of variance (ANOVA) with between-factor, *Group* (young and elderly group), and within-factor, *Task* (NC vs AC), were used to investigate the main effects of *Group* and *Task* and the interaction between *Group* and *Task*. Pairwise comparisons were performed when a significant effect was observed. Statistical significance was set up at p<.05.

## Results

**Fig 3:**
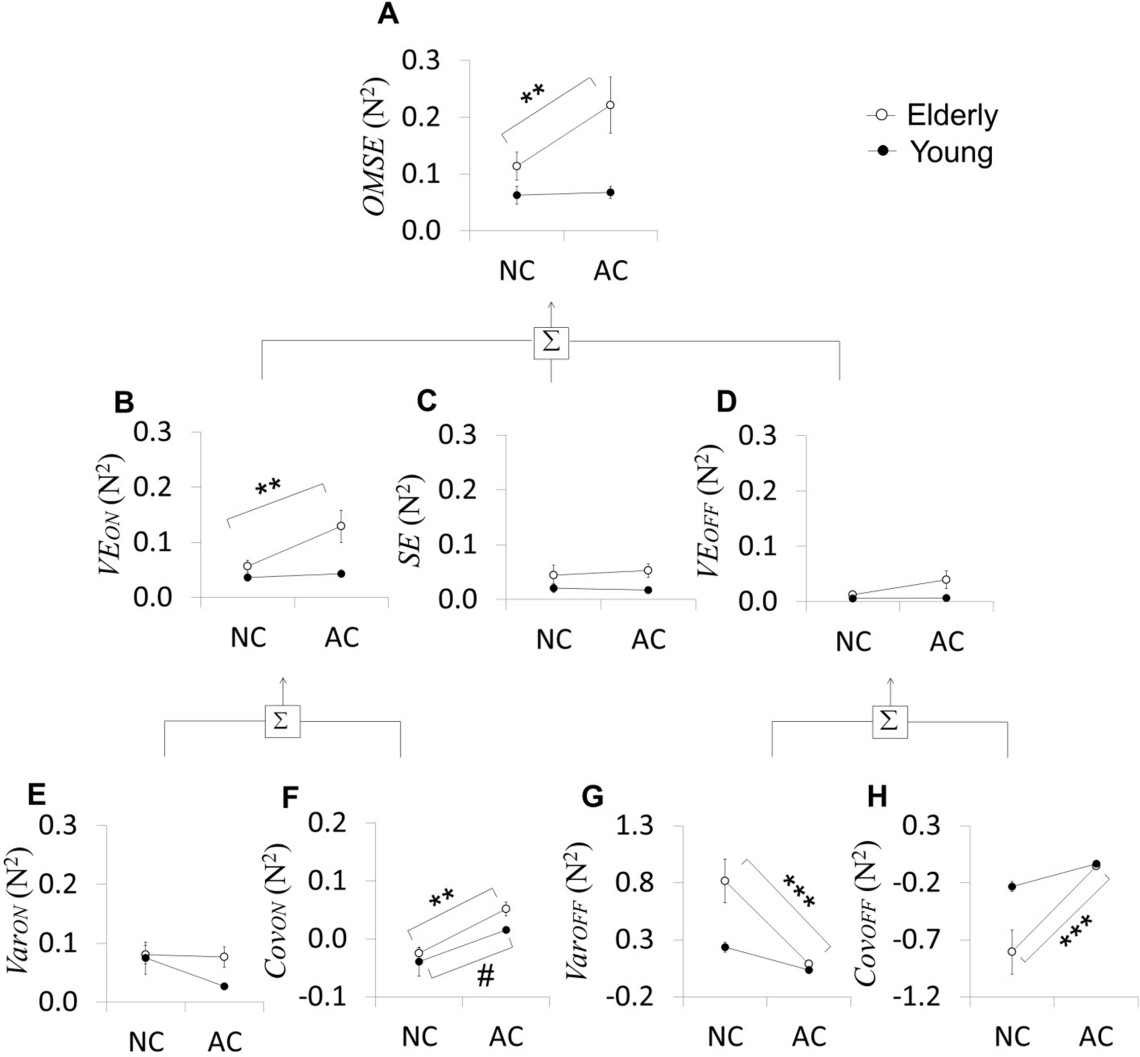
Hierarchical organization of multi-finger force. Overall mean squared error (*OMSE*) is shown in the top panel (A). At the VF level, the *OMSE* was decomposed into online variable error (*VE*_*ON*_) (B), systematic error (*SE*) (C), and offline variable error (*VE*_*OFF*_) (D). At IF level, both *VE*_*ON*_ and *VE*_*OFF*_ are further decomposed into *Var*_*ON*_ (E) & *Cov*_*ON*_ (F) and *Var*_*OFF*_ (G) & *Cov*_*OFF*_ (H), respectively. Error bars represent s.e.m. across subjects. Asterisks (*) and pound signs (#) indicate statistical significance of pair-wise comparisons between NC and AC tasks for the elderly group and the young group, respectively (* and # p<0.05, ** p<0.01, and *** p<0.001).

We analyzed multi-finger actions in a hierarchical manner as VF actions at the higher level and IF actions at the lower level (Fig 2). Several dependent variables were quantified from VF and IF actions using the HVD model (Fig 3).

### Effects of aging on VF actions

The elderly group showed greater *OMSE* during the AC task compared to the NC task while the young group did not show a difference between AC and NC. T/his result suggests that the aging is associated with deterioration in overall motor performance when an additional task constraint was required. This finding was supported by a significant interaction (*Group* X *Task*) (F_1,28_ = 6.049, p = 0.021), along with a significant *Group* effect (F_1,28_ = 7.539; p = 0.011) and a significant *Task* effect (F_1,28_ = 7.346, p = 0.012). The pair-wise comparisons showed that *OMSE* of the elderly group during AC task was significantly greater than NC task (p = 0.001) while *OMSE* of the young group did not differ between tasks. (Fig 3A).

At the VF level, the HVD model decomposes *OMSE* into *VE*_*ON*_ (inverse of *consistency*), *VE*_*ON*_ (inverse of *repeatability*) and *SE* (inverse of *estimability*). The elderly group showed greater *VE*_*ON*_ during the AC task compared to the NC task while the young group did not show a difference between AC and NC. This result indicates that the elderly group deteriorated *consistency* when the additional constraint is imposed to the motor task while the young group did not change *consistency*. However, both group showed unchanged *VE*_*ON*_ and *SE* between tasks. This result indicates that both group performed the both tasks at the similar level of performance in terms of *consistency* and *estimability*. The results suggest that the aging is associated with performance deterioration only in online control when an additional task constraint was required. The findings were supported by a significant interaction (*Group* X *Task*) on *VE*_*ON*_ (F_1,28_ = 5.721, p = 0.024) along with a significant *Group* effect (F_1,28_ = 7.293, p = 0.012) and a significant *Task* effect (F_1,28_ = 8.532, p = 0.007). However, there was no significant interaction on either *VE*_*OFF*_ (F_1,28_ = 2.498, p = 0.126) or *SE* (F_1,28_ = 0.767, p = 0.389) along with no significant *Task* effect (*VE*_*OFF*_: F_1,28_ = 3.159, p = 0.087; *SE*: F_1,28_ = 0.118, p = 0.734) and a significant *Group* effect in *VE*_*OFF*_ (*VE*_*OFF*_: F_1,28_ = 4.546, p = 0.042; *SE*: F_1,28_ = 3.602, p = 0.068). The pair-wise comparisons showed that *VE*_*ON*_ of the elderly group at AC task was significantly greater than NC task (p = 0.004) while *VE*_*ON*_ of the young group did not differ between tasks (Fig 3B).

### Effects of aging on IF actions

In online control of IF actions, although young group showed a trend of smaller *Var*_*ON*_ at AC task compared to NC task, both elderly and young groups showed significantly unchanged *Var*_*ON*_ between AC and NC tasks. However, both groups showed significantly greater *Cov*_*ON*_ at AC task compared to NC task. This result indicates that both group performed the both task within the similar *workspace* in online control, but deteriorated *synergy* in online control when the additional constraint is imposed to the motor task. However, in offline IF actions, the elderly group showed significantly smaller *Var*_*OFF*_ and greater *Cov*_*OFF*_ during the AC task than the NC task, while the young group showed no significant difference between AC and NC even though there was a trend of smaller *Var*_*OFF*_ and greater *Cov*_*OFF*_ during the AC task than the NC task. This result indicates that the elderly group decreased *workspace* and improved *synergy* in offline control when the additional constraint is imposed to the motor task. However, the young group performed the both task within the similar *workspace* with the similar *synergy* in offline control. Interestingly, these results indicate the aging is mostly associated with the deterioration in offline control of IF actions, while the aging is associated with the deterioration in online control of VF actions. These findings are supported by significant interactions in *Var*_*OFF*_ and *Cov*_*OFF*_ (*Var*_*ON*_: F_1,28_ = 1.774, p = 0.194; *Cov*_*ON*_: F_1,28_ = 0.506, p = 0.483; *Var*_*OFF*_: F_1,28_ = 6.440, p = 0.017; *Cov*_*OFF*_: F_1,28_ = 6.755, p = 0.015) along with significant *Task* effects in *Cov*_*ON*_, *Var*_*OFF*_, and *Cov*_*OFF*_ (*Var*_*ON*_: F_1,28_ = 2.413, p = 0.132; *Cov*_*ON*_: F_1,28_ = 19.355, p<0.001; *Var*_*OFF*_: F_1,28_ = 20.059; p<0.001; *Cov*_*OFF*_: F_1,28_ = 20.347, p<0.001) and significant *Group* effects in *Var*_*OFF*_ and *Cov*_*OFF*_ (*Var*_*ON*_: F_1,28_ = 1.692, p = 0.204; *Cov*_*ON*_: F_1,28_ = 2.476, p = 0.127; *Var*_*OFF*_: F_1,28_ = 9.327, p = 0.005; *Cov*_*OFF*_: F_1,28_ = 8.830; p = 0.006). The pair-wise comparisons showed that, in online control, *Cov*_*ON*_ of both group at AC task was significantly greater as compared to NC task (Young group: p = 0.013 and Elderly group: p = 0.001) (Fig 3F). In offline control, *Var*_*OFF*_ of the elderly group was significantly smaller at AC task as compared to NC task (p<0.001) and *Cov*_*OFF*_ of the elderly group at AC task was significantly greater as compared to NC task (p<0.001).

## Discussion

Our study investigated aging-related changes in the hierarchical organization of multi-finger force control during two-finger pressing tasks, which induced *COI* problem to the CNS. We employed the HVD model which quantified several distinct aspects of hierarchically organized multi-finger actions of VF actions at the higher level and IF actions at the lower level. We hypothesized that the aging would be associated with the deterioration in VF and IF actions when an additional constraint as the *COI* problem was introduced to the CNS in our experiment. The hypothesis was largely confirmed by the experimental results. However, interestingly, aging affected VF and IF actions differently for online and offline controls. At the VF level, the elderly group showed deterioration of online VF control when an additional task constraint causing a *COI* problem was introduced, while the young group did not change their performance in online control of VF. Interestingly, at the IF level, the elderly group used smaller offline *workspace* (i.e. *Var*_*OFF*_) and smaller offline *synergy* (i.e. inverse of *Cov*_*OFF*_) [13] when the additional constraint was introduced. However, the young group showed the unchanged *workspace* or *synergy* regardless of the task constraints.

Overall, we found that aging is associated with the deterioration of multi-finger actions when the CNS faces the *COI* problem. According to the principle of superposition which was originally introduced in robotics [15] and later confirmed in human hand experiments [16, 24], human multi-finger actions can be decomposed into sub-actions such as force- and moment-stabilizing actions that are controlled independently by separate controllers. The principle implies that there should be no interference between force-stabilizing and moment-stabilizing task. Indeed, the young group in the current study showed that there was no performance difference between NC (i.e. force-stabilizing task) and AC task (i.e. force + moment-stabilizing task), indicating that moment-stabilizing task did not interference with force-stabilizing task. However, the elderly group showed that the deteriorated consistency during AC task, indicating that aging might have led to the deterioration of the CNS’s abilities to independently control two concurrent sub-tasks (i.e. force- and moment-stabilizing tasks) with conflicts during AC task. Note that we quantified performance of force-stabilizing control for both NC and AC tasks without considering the moment arms of finger tips. Although there was no redundancy in AC task in terms of finger forces, the task could be redundant with varying moment arms. Thus, it may be possible that the young group has ability to perform AC task at the similar level of performance for NC task by changing moment arms of finger tips, which warrants the further study.

We found that aging is associated with deteriorated overall motor performance (i.e. inverse of *OMSE*) that was contributed by deteriorated *consistency* of force control in online control (i.e. inverse of *VE*_*ON*_) during AC task compared with the NC task. This result indicates that decline in the CNS’s ability to produce consistent actions when elderly face the *COI* problem is mainly due to declines in force control in online control, not offline control or systematic errors. Several previous studies reported that aging leads to increase variability of force produced during hand actions [25–27]. In particular, Vaillancourt and Newell (27) asked subjects to produce finger force to match a simpler constant target force and a more complex sinusoidal target force. They found that the elderly subjects showed declined performance in a sinusoidal target force control as compared to the constant force control. In our study, AC task provided an additional task constraint while introducing the *COI* problem, which is similar to the sine wave force control in Vaillancourt and Newell (27) in terms of its greater task complexity and cognitive load to the CNS. These two studies provide converging evidence that aging leads deficits in performance of online control when the task is more complex and requires greater cognitive load to the CNS of the elderly.

Intriguingly, the *COI* problem introduced in our study negatively affected virtual finger actions at the higher level only in online control, while the same problem affected individual finger actions at the lower level only in offline control. Our study also found that aging is associated with the decreased offline *synergy* (i.e. increased *Cov*_*OFF*_) when the additional torque constraint was introduced. This result indicates that the CNS of the elderly changes synergistic actions between fingers by generating different sharing patterns of IF forces over multiple trials. According to the principle of minimization of secondary moments [28], the CNS generates the sharing pattern (i.e. a combination of percentages of total force generated by each finger) during a constant force production task in such a way that the moment of force with respect to the longitudinal axis of the hand is minimized. In two finger pressing task, this principle implies that covariance between two finger forces should be almost zero in order to minimize the moment generated by two finger forces and minimize performance error because positive and negative covariances between finger forces contribute to the force control and torque control errors, respectively. Although moment arms of finger tips were not involved in our analysis, covariance closed to zero during NC task implies that each finger force were produced in the similar magnitude. Thus, in our study, the principle of minimization of secondary moments holds only in the young group who showed the unchanged covariance with the additional constraint, which is also consistent with the previous finding [20].

One of the main factors that contributes the minimum moments of force is the enslaving effect (force production by unintended fingers). Enslaving is a phenomenon that presents forces produced by fingers not explicitly involved in a finger-pressing task [29, 30]. The phenomenon occurs because of both central factors such as overlapping cortical representations for adjacent fingertips in sensory cortex and peripheral factors such as shared muscles and tendinous connections of fingers [29, 31, 32]. Several studies have been performed to investigate the effects of aging on the enslaving effects [3, 5, 33–39]. It was reported that there was a lower indices of finger force enslaving in the elderly as compared to young subjects [5], strength training in the elderly led to higher enslaving indexes [38], and fatigue did not change the enslaving indexes [39]. In addition, previous studies suggest that aging is associated with a decline in the number of neurons especially alpha-motoneurons [33, 34], and the decreased average size of muscle fibers [35, 36], leading to weaker enslaving effects [3, 5, 37]. Thus, the decreased enslaving effects after aging might have caused changes in offline *synergy* with an additional torque constraint in our study.

## Limitation

During multi-finger actions, individual fingers show phenomena of mutual dependence due to the enslaving effects [40, 41]. Previously, the hypothetical CNS commands to individual fingers (i.e. finger force modes) have been calculated [42] from estimation of couplings between individual finger forces (i.e. finger enslaving, [30]). The analysis of our experimental data in the finger mode space might have provided additional insights into the actions fingers and interactions between them. However, application of the mode analysis to our study might have been challenging because our study employed two different tasks for NC and AC, and AC task is associated with a different set of task constraints and the finger force mode analysis depends on task constraints. A moment-stabilizing task requires certain levels of finger forces that are required for keeping the resultant moment of force as compared to the force-stabilizing task. In addition, a previous study has shown that the dynamic process of finger force production may be associated with the changes in the enslaving between fingers [43]. Due to these analytical challenges, the analysis of our study was limited in the finger force space. However, if one assumes that the enslaving between fingers is constant in our study, the main findings of our study should still stay hold, specifically those differences observed between NC and AC tasks.

Our study could have employed another task that requires a constant moment of or zero moment of force in order to systematically compare three tasks that require a constant force (NC task), a constant moment of force, and both constant force and moment (AC task). However, we did use the constant moment task in our study because, theoretically, subjects could produce a constant moment without producing any finger forces. Previous studies have shown that force variability and associated with force magnitude [44, 45], which could lead to different level of force variability between groups. In addition, our experimental design was more focused on a within-subject design to investigate how each group performed the task in two different conditions. We have tried to minimize this potential issue by setting the same target force level within two task conditions.

## References

[1] Boatright JR, Kiebzak GM, O'Neil DM, Peindl RD. Measurement of thumb abduction strength: normative data and a comparison with grip and pinch strength. The Journal of hand surgery. 1997;22(5):843–8. doi: 10.1016/S0363-5023(97)80079-2 . 9330143 10.1016/S0363-5023(97)80079-2

[2] Hughes S, Gibbs J, Dunlop D, Edelman P, Singer R, Chang RW. Predictors of decline in manual performance in older adults. Journal of the American Geriatrics Society. 1997;45(8):905–10. . 9256839 10.1111/j.1532-5415.1997.tb02957.x

[3] Shinohara M, Latash ML, Zatsiorsky VM. Age effects on force produced by intrinsic and extrinsic hand muscles and finger interaction during MVC tasks. Journal of applied physiology. 2003;95(4):1361–9. doi: 10.1152/japplphysiol.00070.2003 12626484 10.1152/japplphysiol.00070.2003

[4] Winegard KJ, Hicks AL, Vandervoort AA. An evaluation of the length–tension relationship in elderly human plantarflexor muscles. The Journals of Gerontology Series A: Biological Sciences and Medical Sciences. 1997;52(6):B337–B43. 10.1093/gerona/52a.6.b337 9402935

[5] Shinohara M, Li S, Kang N, Zatsiorsky VM, Latash ML. Effects of age and gender on finger coordination in MVC and submaximal force-matching tasks. Journal of applied physiology. 2003;94(1):259–70. doi: 10.1152/japplphysiol.00643.2002 . 12391031 10.1152/japplphysiol.00643.2002

[6] Cole KJ, Rotella DL, Harper JG. Mechanisms for age-related changes of fingertip forces during precision gripping and lifting in adults. The Journal of neuroscience: the official journal of the Society for Neuroscience. 1999;19(8):3238–47. . 10191336 10.1523/JNEUROSCI.19-08-03238.1999 PMC6782297

[7] Zatsiorsky VM. Kinematics of human motion, Human Kinetics Urbana Champaign 1998.

[8] Arbib MA. Coordinated control programs for movements of the hand. Hand function and the neocortex. 1985:111–29.

[9] Baud-Bovy G, Soechting JF. Two virtual fingers in the control of the tripod grasp. Journal of Neurophysiology. 2001;86(2):604–15. doi: 10.1152/jn.2001.86.2.604 11495936 10.1152/jn.2001.86.2.604

[10] Shim JK, Latash ML, Zatsiorsky VM. Prehension synergies in three dimensions. Journal of neurophysiology. 2005;93(2):766–76. doi: 10.1152/jn.00764.2004 15456799 10.1152/jn.00764.2004 PMC2827185

[11] Shim JK, Latash ML, Zatsiorsky VM. Prehension synergies: trial-to-trial variability and principle of superposition during static prehension in three dimensions. Journal of neurophysiology. 2005;93(6):3649–58. doi: 10.1152/jn.01262.2004 15728759 10.1152/jn.01262.2004 PMC2827186

[12] Koh K, Kwon HJ, Yoon BC, Cho Y, Shin JH, Hahn JO, et al The role of tactile sensation in online and offline hierarchical control of multi-finger force synergy. Experimental brain research. 2015;233(9):2539–48. doi: 10.1007/s00221-015-4325-6 . 26019011 10.1007/s00221-015-4325-6

[13] Koh K, Kwon HJ, Park YS, Kiemel T, Miller RH, Kim YH, et al Intra-auditory integration improves motor performance and synergy in an accurate multi-finger pressing task. Frontiers in Human Neuroscience. 2016;10 doi: 10.3389/fnhum.2016.00260 27375457 10.3389/fnhum.2016.00260 PMC4896966

[14] Latash ML. The bliss (not the problem) of motor abundance (not redundancy). Experimental brain research. 2012;217(1):1–5. doi: 10.1007/s00221-012-3000-4 ; PubMed Central PMCID: PMC3532046. 22246105 10.1007/s00221-012-3000-4 PMC3532046

[15] Arimoto S, Tahara K, Yamaguchi M, Nguyen PTA, Han M-Y. Principles of superposition for controlling pinch motions by means of robot fingers with soft tips. Robotica. 2001;19(01):21–8.

[16] Zatsiorsky VM, Latash ML, Gao F, Shim JK. The principle of superposition in human prehension. Robotica. 2004;22(2):231–4. doi: 10.1017/S0263574703005344 ; PubMed Central PMCID: PMC2827859. 20186284 10.1017/S0263574703005344 PMC2827859

[17] Vaportzis E, Georgiou-Karistianis N, Stout JC. Dual task performance in normal aging: a comparison of choice reaction time tasks. PloS one. 2013;8(3):e60265 doi: 10.1371/journal.pone.0060265 ; PubMed Central PMCID: PMC3605385. 23555937 10.1371/journal.pone.0060265 PMC3605385

[18] Hollman JH, Kovash FM, Kubik JJ, Linbo RA. Age-related differences in spatiotemporal markers of gait stability during dual task walking. Gait & posture. 2007;26(1):113–9. doi: 10.1016/j.gaitpost.2006.08.005 . 16959488 10.1016/j.gaitpost.2006.08.005

[19] Huxhold O, Li SC, Schmiedek F, Lindenberger U. Dual-tasking postural control: aging and the effects of cognitive demand in conjunction with focus of attention. Brain research bulletin. 2006;69(3):294–305. doi: 10.1016/j.brainresbull.2006.01.002 . 16564425 10.1016/j.brainresbull.2006.01.002

[20] Zhang W, Scholz JP, Zatsiorsky VM, Latash ML. What do synergies do? Effects of secondary constraints on multidigit synergies in accurate force-production tasks. J Neurophysiol. 2008;99(2):500–13. doi: 10.1152/jn.01029.2007 ; PubMed Central PMCID: PMC2827038. 18046000 10.1152/jn.01029.2007 PMC2827038

[21] Shinohara M, Scholz JP, Zatsiorsky VM, Latash ML. Finger interaction during accurate multi-finger force production tasks in young and elderly persons. Experimental brain research. 2004;156(3):282–92. doi: 10.1007/s00221-003-1786-9 . 14985892 10.1007/s00221-003-1786-9

[22] Maylor EA, Wing AM. Age differences in postural stability are increased by additional cognitive demands. The journals of gerontology Series B, Psychological sciences and social sciences. 1996;51(3):P143–54. . 8620354 10.1093/geronb/51b.3.p143

[23] Verhaeghen P, Steitz DW, Sliwinski MJ, Cerella J. Aging and dual-task performance: a meta-analysis. Psychology and aging. 2003;18(3):443–60. doi: 10.1037/0882-7974.18.3.443 . 14518807 10.1037/0882-7974.18.3.443

[24] Shim JK, Latash ML, Zatsiorsky VM. Prehension synergies: trial-to-trial variability and principle of superposition during static prehension in three dimensions. J Neurophysiol. 2005;93(6):3649–58. doi: 10.1152/jn.01262.2004 ; PubMed Central PMCID: PMC2827186. 15728759 10.1152/jn.01262.2004 PMC2827186

[25] Galganski ME, Fuglevand AJ, Enoka RM. Reduced control of motor output in a human hand muscle of elderly subjects during submaximal contractions. J Neurophysiol. 1993;69(6):2108–15. doi: 10.1152/jn.1993.69.6.2108 . 8350134 10.1152/jn.1993.69.6.2108

[26] Laidlaw DH, Bilodeau M, Enoka RM. Steadiness is reduced and motor unit discharge is more variable in old adults. Muscle & nerve. 2000;23(4):600–12. 10716772 10.1002/(sici)1097-4598(200004)23:4<600::aid-mus20>3.0.co;2-d

[27] Vaillancourt DE, Newell KM. Aging and the time and frequency structure of force output variability. Journal of applied physiology. 2003;94(3):903–12. doi: 10.1152/japplphysiol.00166.2002 . 12571125 10.1152/japplphysiol.00166.2002

[28] Li Z-M, Latash M, Zatsiorsky V. Force sharing among fingers as a model of the redundancy problem. Experimental brain research. 1998;119(3):276–86. 9551828 10.1007/s002210050343

[29] Schieber MH. Individuated finger movements of rhesus monkeys: a means of quantifying the independence of the digits. Journal of neurophysiology. 1991;65(6):1381–91. doi: 10.1152/jn.1991.65.6.1381 1875247 10.1152/jn.1991.65.6.1381

[30] Zatsiorsky VM, Li ZM, Latash ML. Enslaving effects in multi-finger force production. Experimental brain research. 2000;131(2):187–95. . 10766271 10.1007/s002219900261

[31] Schieber MH, Hibbard LS. How somatotopic is the motor cortex hand area? SCIENCE-NEW YORK THEN WASHINGTON-. 1993:489-. 10.1126/science.8332915 8332915

[32] Kilbreath S, Gandevia S. Limited independent flexion of the thumb and fingers in human subjects. The Journal of Physiology. 1994;479(Pt 3):487. 7837104 10.1113/jphysiol.1994.sp020312 PMC1155766

[33] Tomlinson BE, Irving D. The numbers of limb motor neurons in the human lumbosacral cord throughout life. Journal of the neurological sciences. 1977;34(2):213–9. . 925710 10.1016/0022-510x(77)90069-7

[34] Morrison JH, Hof PR. Life and death of neurons in the aging brain. Science. 1997;278(5337):412–9. . 9334292 10.1126/science.278.5337.412

[35] Bemben MG. Age-related alterations in muscular endurance. Sports medicine. 1998;25(4):259–69. . 9587183 10.2165/00007256-199825040-00004

[36] Kirkendall DT, Garrett WE Jr. The effects of aging and training on skeletal muscle. The American journal of sports medicine. 1998;26(4):598–602. doi: 10.1177/03635465980260042401 . 9689386 10.1177/03635465980260042401

[37] Oliveira MA, Hsu J, Park J, Clark JE, Shim JK. Age-related changes in multi-finger interactions in adults during maximum voluntary finger force production tasks. Human movement science. 2008;27(5):714–27. doi: 10.1016/j.humov.2008.04.005 ; PubMed Central PMCID: PMC2637388. 18762348 10.1016/j.humov.2008.04.005 PMC2637388

[38] Olafsdottir HB, Zatsiorsky VM, Latash ML. The effects of strength training on finger strength and hand dexterity in healthy elderly individuals. Journal of applied physiology. 2008;105(4):1166–78. doi: 10.1152/japplphysiol.00054.2008 ; PubMed Central PMCID: PMC2576040. 18687981 10.1152/japplphysiol.00054.2008 PMC2576040

[39] Singh T, Zatsiorsky VM, Latash ML. Contrasting effects of fatigue on multifinger coordination in young and older adults. Journal of applied physiology. 2013;115(4):456–67. doi: 10.1152/japplphysiol.00375.2013 ; PubMed Central PMCID: PMC3742945. 23743395 10.1152/japplphysiol.00375.2013 PMC3742945

[40] Kilbreath SL, Gandevia SC. Limited independent flexion of the thumb and fingers in human subjects. J Physiol. 1994;479 Pt 3):487–97. ; PubMed Central PMCID: PMC1155766. 7837104 10.1113/jphysiol.1994.sp020312 PMC1155766

[41] Schieber MH, Gardinier J, Liu J. Tension distribution to the five digits of the hand by neuromuscular compartments in the macaque flexor digitorum profundus. The Journal of neuroscience: the official journal of the Society for Neuroscience. 2001;21(6):2150–8. . 11245699 10.1523/JNEUROSCI.21-06-02150.2001 PMC6762629

[42] Danion F, Schoner G, Latash ML, Li S, Scholz JP, Zatsiorsky VM. A mode hypothesis for finger interaction during multi-finger force-production tasks. Biol Cybern. 2003;88(2):91–8. doi: 10.1007/s00422-002-0336-z . 12567224 10.1007/s00422-002-0336-z

[43] Martin JR, Latash ML, Zatsiorsky VM. Interaction of finger enslaving and error compensation in multiple finger force production. Experimental brain research. 2009;192(2):293–8. doi: 10.1007/s00221-008-1615-2 ; PubMed Central PMCID: PMC2648126. 18985331 10.1007/s00221-008-1615-2 PMC2648126

[44] Schmidt RA, Zelaznik H, Hawkins B, Frank JS, Quinn JT Jr. Motor-output variability: a theory for the accuracy of rapid motor acts. Psychological review. 1979;47(5):415–51. . 504536

[45] Jones KE, Hamilton AF, Wolpert DM. Sources of signal-dependent noise during isometric force production. Journal of neurophysiology. 2002;88(3):1533–44. doi: 10.1152/jn.2002.88.3.1533 . 12205173 10.1152/jn.2002.88.3.1533

